# Punishment as a scarce resource: a potential policy intervention for managing incarceration rates

**DOI:** 10.3389/fpsyg.2023.1157460

**Published:** 2023-05-05

**Authors:** Eyal Aharoni, Eddy Nahmias, Morris B. Hoffman, Sharlene Fernandes

**Affiliations:** ^1^Georgia State University, Atlanta, GA, United States; ^2^Independent Researcher, Denver, CO, United States

**Keywords:** criminal sentences, cost—benefit, decision making, punishment, criminal justice policy, cost disclosure, cost discounting, mass incarceration

## Abstract

Scholars have proposed that incarceration rates might be reduced by a requirement that judges justify incarceration decisions with respect to their operational costs (e.g., prison capacity). In an Internet-based vignette experiment (*N* = 214), we tested this prediction by examining whether criminal punishment judgments (prison vs. probation) among university undergraduates would be influenced by a prompt to provide a justification for one's judgment, and by a brief message describing prison capacity costs. We found that (1) the justification prompt alone was sufficient to reduce incarceration rates, (2) the prison capacity message also independently reduced incarceration rates, and (3) incarceration rates were most strongly reduced (by about 25%) when decision makers were asked to justify their sentences with respect to the expected capacity costs. These effects survived a test of robustness and occurred regardless of whether participants reported that prison costs should influence judgments of incarceration. At the individual crime level, the least serious crimes were most amenable to reconsideration for probation. These findings are important for policymakers attempting to manage high incarceration rates.

## Introduction

The U.S. is among the leaders in rates of incarceration, incarcerating more than 500 people per 100,000 (Carson, 2021; Statistica Research Department, 2023). Overcrowded conditions, especially in state prisons and county jails, have repeatedly been found to violate inmates' rights against cruel and unusual punishment (e.g., Brown v. Plata, 2011). Efforts to relieve state prison capacity (e.g., Public Safety Realignment Act of 2011 Implementation Plan, 2011) often transfer the burden to local jails (e.g., see Kang-Brown et al., 2021). Despite these efforts, the special deterrent effects of incarceration remain controversial, as many inmates are more dangerous coming out of jail or prison than going in Stemen (2017).

Multiple factors likely contribute to these high incarceration rates, including increased crime, better detection methods, stricter sentencing laws, socio-economic inequality, and the legacy of racist incarceral institutions (see Alexander, 2010). Some of these factors depend on the psychology of decision making, stimulating a growing body of research on judicial decision-making bias (e.g., Guthrie et al., 2000; Englich et al., 2006; Danziger et al., 2011). One underappreciated contributor to high incarceration rates is that the individuals who make sentencing decisions in virtually every American jurisdiction—trial court judges—are encouraged to justify incarceration with respect to its societal benefits but not to its costs. Those costs, including the cost of building and operating prisons and jails—money that could otherwise be invested into other services (e.g., mental health, vocational training, and policing)—are offloaded to other branches of government (Bierschbach and Bibas, 2017).

This asymmetry in the choice architecture that judges face can lead to very high rates of punishment. Research has shown that when prosecutors and judges are insulated from information about sentencing costs, their recommended and imposed prison terms are as much as 30% longer than those made when the costs are disclosed (Aharoni et al., 2021, 2022, see also Rachlinski et al., 2013). At least one of these studies has suggested that participants do not appear to be aware of this bias toward assuming benefits but not costs of incarceration (Aharoni et al., 2021). This asymmetry occurs, we theorize, because judges are trained and expected to *maximize* the societal benefits of incarceration, rather than to *optimize* those benefits by balancing them against incarceration's costs (see Aharoni et al., 2020). This asymmetry may have its roots in the doctrine of separation of powers—legislatures, not judges, decide on sentencing ranges and how many prisons to build. It may also be influenced by retributive justifications for punishment, which focus on what the offender deserves and not on the costs and benefits of punishment (Carlsmith, 2006; Aharoni and Fridlund, 2012). Nevertheless, whether judges are consciously or unconsciously ignoring the costs of criminal punishment, it likely has a direct, aggravating effect on incarceration rates (Bierschbach and Bibas, 2017; Aharoni et al., 2022).

To remediate this bias toward incarceration, without violating the separation of powers, some scholars have argued for policy mechanisms that would encourage trial judges to internalize sentencing costs by making those costs explicit. Notably, Jonson et al. (2014) proposed the imposition of a “soft cap” on the number of felons who could be sentenced to prison each year (Jonson et al., 2014; see also Bierschbach and Bibas, 2017). If the prison capacity is exceeded, taxpayers would have to pay for the additional prison beds needed, so judges would be required to provide a written justification explaining why their sentencing determination is worth the additional cost to taxpayers. The prediction—which we sought to test—is that simply asking judges to justify overages in prison capacity should reduce their reliance on incarceration by encouraging them to more deeply process decision-relevant factors, such as the cost to taxpayers of incarceration.

Importantly, the policy does not prevent judges from exceeding capacity—and taxpayers are on the hook for each sentence whether the judge is aware of it or not—so the policy does not impose any new material constraint on sentencing. It simply reminds judges that their decisions represent an inherent tradeoff between incarceration and other public interests. In this way, the policy would attempt to change the judge's choice architecture by drawing attention to additional factors that might be relevant to their decision. Since trial judges are already expected to provide justifications for many of their decisions (e.g., in civil and criminal bench trials), asking them to justify these decisions with respect to their consequences would require minimal, if any, statutory revision and may even be handled at the local rule level.

From a retributive perspective, we might not expect sentencing judges to care about correctional resources. They might be expected to sentence based on what offenders deserve for their crimes. But the desire for retribution should not imply complete insensitivity to all negative consequences. To illustrate, one-third to one-half of prison inmates are serving sentences for non-violent offenses (Austin et al., 2016), and the correctional resources required to incarcerate these offenders are roughly the same as those required to incarcerate violent offenders. If, as we have shown elsewhere, people's motivations for punishment are influenced by the immediate salience of the punishments' expected costs and benefits (Aharoni et al., 2020, 2021, 2022), and prison capacity represents a cost of incarceration, then it seems plausible that judges will reduce their reliance on incarceration, perhaps for the less serious offenders, when evaluated in the presence of capacity information.

Real-world applications of this policy proposal are conspicuously lacking (for an exception, see the MN Enabling Act; Frase, 1991), but evidence that such an intervention substantially reduces incarceration would be consequential for virtually all jurisdictions struggling with overcrowding and over-incarceration. Ultimately, all sentencing decisions are choices to trade off scarce taxpayer resources. To the extent that judges ignore these constraints, they are applying a *de facto* policy to punish at any cost. Therefore, understanding how awareness of these constraints affects decisions to incarcerate has direct importance for all jurisdictions.

If judges rely on similar decision making strategies as other people, then a test of this question among non-experts should provide a crucial proof of concept for research on judicial decision making. Although laypersons' judgments do not necessarily generalize to judgments by professional judges, previous research has shown striking parallels in decision processes between judges and laypersons, in terms of convictions rates (see Robbennolt, 2005), and psychological processes (see Guthrie et al., 2000; Teichman and Zamir, 2014; Miller, 2019), and research on cost framing in particular has observed similar effects among legal experts, community members, and university students (Aharoni et al., 2019, 2020, 2021, 2022). Those studies primarily tested the effects of information about the financial cost of incarceration on sentence length determinations. The present study is the first to examine placement decisions (e.g., prison vs. probation), and to test the effect of policy proposals that require decision makers to justify their prison sentence recommendations and to do so with respect to operational costs. Understanding the punishment attitudes of non-experts in this context is also important because these attitudes may motivate and shape civic engagement. For instance, these attitudes may influence how citizens vote for candidates or policies relevant to the criminal justice system and its outcomes.

Compared to other research methods, experiments have the unique advantage of enabling investigators to disentangle various causal influences on sentencing behavior to measure the specific impact of each factor. We conducted a survey experiment with laypersons to test the effects of a soft prison cap policy on punishment judgments before and after a requirement to justify one's judgment to sentence convicted offenders to prison. Specifically, we tested (1) whether and to what extent a brief informational message about prison capacity reduces incarceration rates. We also tested (2) whether such an effect is compounded by a requirement to justify one's decision to incarcerate. Finally, we tested (3) whether and how the impact of these factors depends on the type of offense (e.g., drug trafficking vs. murder), on self-reported measures of whether costs should influence these judgments, and on basic demographic information about the decision maker (e.g., political ideology). We hypothesized that the instruction to justify one's prison sentences, and the instruction to do so with respect to prison capacity considerations, would reduce incarceration rates, both independently and interactively. We also predicted that these effects would be strongest for less serious crimes.

## Methods

### Participants

Participants were 313 university undergraduate students participating for course credit in one of three introductory courses in Psychology, Philosophy, or Political Science. To be eligible to participate, participants were informed that they must be at least age 18, be fluent in English, and be a U.S. citizen. Based on our a priori exclusion criteria, 96 were excluded for failing to complete the survey (44 of these did not start the survey, and the other 52 dropped out after completing the primary dependent measures); three more were excluded for failing a multiple-choice attention check question “What are the colors of the American flag?”. For the remaining 214 participants, the sample was 56.5% female, 38.3% male, and 5.1% other or preferred not to answer, with ages from 18 to 42 years, *M* = 21.73, *SE* = 0.32. Race and ethnicity data were not collected, but the university's undergraduate population is 41% Black, 23% White, 16% Asian, 13% Hispanic, 5% mixed race, 1% unknown, <1% American Indian/Alaska Native, and <1% Native Hawaiian/Pacific Islander (University System of Georgia, 2022).

Our minimum estimated sample size was based on an *a priori* power analysis for detecting a between-subjects interaction between our primary independent variable and a single moderator, assuming: a medium effect size of *f* = 0.30; 1-β = 0.80; α = 0.05; *r* = 0.4 correlation between our repeated measures. This analysis yielded a minimum sample size estimate of 111.

### Design and procedure

Our study followed a 2 (prison capacity information) × 2 (sentencing measure before or after justification prompt) mixed design. Participants were asked to imagine they were a judge making sentencing decisions (prison vs. probation) for nine criminal offenders described in case summaries. Half of the participants were first exposed to an instruction that the prison has reached full capacity, so for every additional offender they sentence to prison, they will be required to provide a written justification for that decision (the treatment condition). The other half received instructions that did not mention prison capacity or a justification requirement (the control condition). Then all participants made initial sentencing decisions for all nine cases (the baseline sentencing measure). We were interested in observing within-person judgment change. But because undergraduates lack the background knowledge likely used by professional judges to benchmark new judgments, we first asked our participants to make initial sentencing decisions for all nine cases (the baseline sentencing measure), providing them with a contextual frame of reference within which to organize their judgments. Then, after all sentencing decisions were submitted, the participants who initially chose prison for a given offender were given that full case text again to refresh their memories and given an opportunity to either justify (and thereby reaffirm) their prison sentence or change it to probation instead (the secondary sentencing measure). This approach affords greater statistical sensitivity than other designs and a more direct test of individual judgment change. A pre-justification and post-justification incarceration rate were calculated for each participant by dividing the number of defendants sentenced to prison by the nine crimes. Post-justification incarceration rates were not calculated for five participants who did not recommend any prison time. Our primary hypotheses were tested using a mixed Analysis of Variance (ANOVA), which permits comparison of between-subject and within-subject factors on incarceration rates (the dependent measure). For exploratory purposes, participants who persisted in their decision to incarcerate received an additional instruction to make a sentence length recommendation for each crime (see Appendix B for the sentence length results.) Several covariates were collected to test for possible moderating influence, including participants' political ideology, age, and gender, and their normative attitudes about whether capacity limits should factor into judicial sentencing decisions. Textual sentencing justifications were subjected to a qualitative analysis of the relative prevalence of each justification type: “retributive,” “utilitarian,” both of these, or none of these (see Appendix B for qualitative analysis). All study procedures were approved by the university's ethical review board and conditioned on informed consent.

### Materials

The nine case summaries (burglary, tax fraud, simple battery, drug trafficking, insurance fraud, assault and battery, armed robbery, aggravated robbery, and murder) were adapted from scenarios reported in Robinson and Kurzban (2007) and Kugler et al. (2013) and selected to cover a broad range of crime types (mean word count = 47; see Appendix A for case scenarios). The survey instructions asked participants to make sentencing decisions for each crime. Half of the participants received an additional instruction to justify any prison sentences with respect to its costs (see Appendix A for survey instructions). The dependent measure presented a dichotomous choice: “Should this offender be incarcerated?” (YES, the offender should be incarcerated vs. NO, the offender should be sentenced to probation).

The wording of the justification prompt differed slightly by condition in order to make the question meaningful for that condition. For the participants who recommended prison following exposure to the capacity information, they were asked to *justify* why their decision to increase prison capacity is worth the additional cost to taxpayers. For the participants who recommended prison in the control condition, they were asked simply to *explain* why they believe the offender should be sentenced to prison instead of probation. Then participants were given an opportunity to either uphold their sentence recommendation and provide a written justification for it, or else change their sentence recommendation in favor of probation.

After these measures, we assessed, using a 7-point Likert-type scale from “strongly disagree” (-3) to “strongly agree” (+3), their agreement with a statement that judges should consider the capacity limits of their prisons when deciding how an offender should be sentenced. Another forced-choice question assessed participants' self-reported philosophical justifications for punishment (retributive vs. utilitarian), adapted from Nadelhoffer et al. (2013). Last, we collected self-reported gender, birth year, political ideology on a scale from “very liberal” (-3) to “very conservative” (+3), and our aforementioned attention check question.^1^ The datasets presented in this study can be found at: https://osf.io/sp983, Open Science Framework.

## Results

### Confirmatory analysis

**Figure 1:**
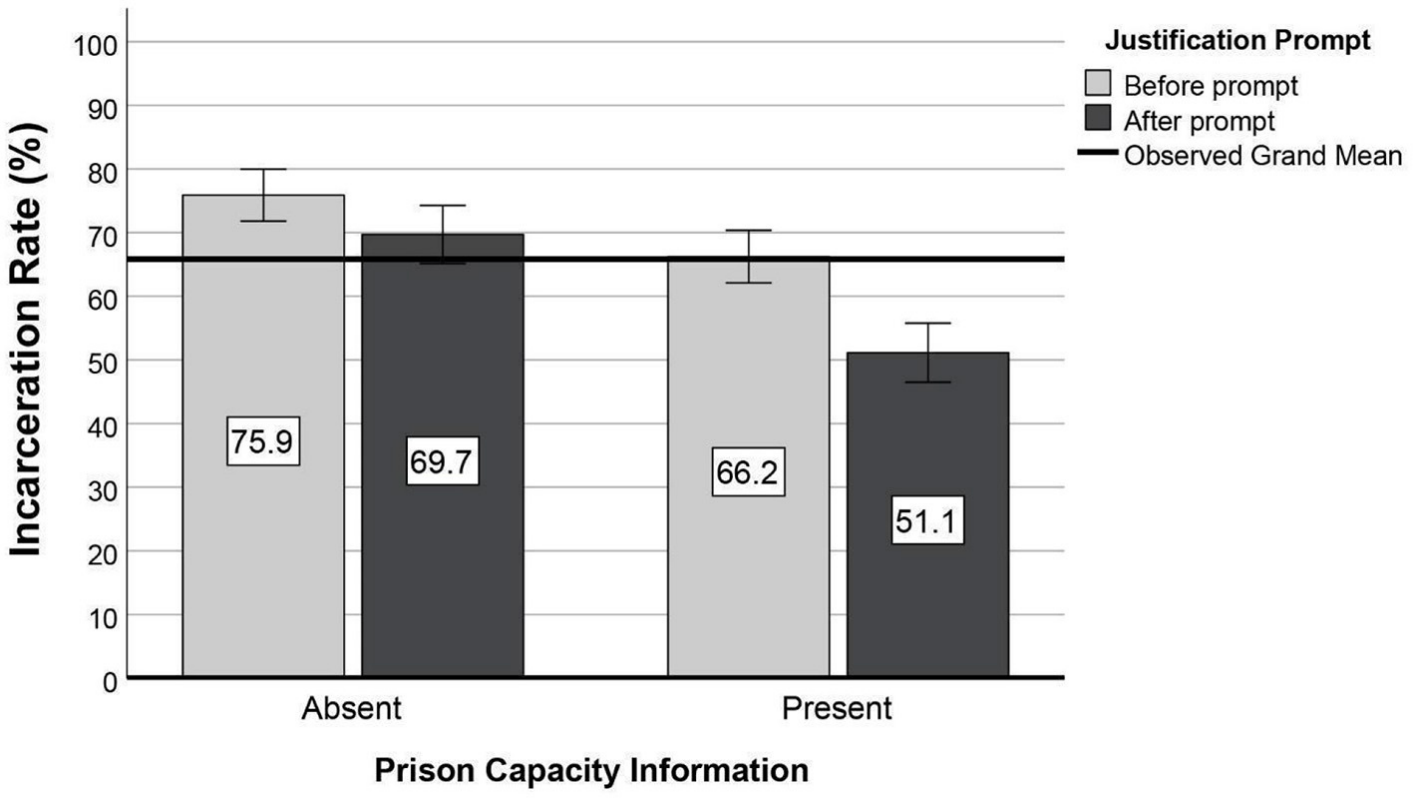
Effects of capacity information and justification prompt on incarceration rates in 214 students across nine crimes. All main effects and interactions are significant at *p* < 0.001. Error bars: 95% *CI*.

We conducted a mixed ANOVA to test the effects of capacity information (between-subjects) and justification prompt (within-subjects) on incarceration rates. Scores on the two sentencing measures were found to have equal variances, as assessed by Levene's test, all *p*'s > 0.10. The model was significant, and all of our predictions were supported. For those who initially recommended incarceration for at least one of the nine cases (97.7% of the sample), simply being asked to either justify their prison sentence or change it to probation reduced the number of defendants sentenced to prison by 10.64% [*M* = 60.42%, *SE* = 1.65, 95% *CI* (57.16, 63.68)] relative to that obtained before the prompt [*M* = 71.06%, *SE* = 1.47, 95% *CI* (68.16, 73.96)], *F*_(1, 207)_ = 84.83, *p* < 0.001, $\eta_{\text{p}}^{2}$ = 0.291. This suggests that a justification prompt alone is sufficient to reduce the incarceration rate. A main effect of capacity information was also obtained, *F*_(1, 207)_ = 23.57, *p* < 0.001, $\eta_{\text{p}}^{2}$ = 0.102. Incarceration rates were 14.12% lower among participants who were exposed to the capacity information [*M* = 58.68%, *SE* = 2.07, 95% *CI* (54.60, 62.77)] than those who were not [*M* = 72.80%, *SE* = 2.04, 95% *CI* (68.78, 76.82)]. These main effects were qualified by an interaction, *F*_(1, 207)_ = 14.89, *p* < 0.001, $\eta_{\text{p}}^{2}$ = 0.067. Pairwise comparisons using Bonferroni correction for multiple comparisons (i.e., four comparisons, where critical *p* = 0.013) revealed that incarceration rates were lowest when participants were exposed both to the capacity information and the justification prompt compared to when they were exposed to the capacity information alone or the justification prompt alone. Indeed, these incarceration rates were 24.76% lower than observed when participants were not exposed to either manipulation. In other words, about one in four prison sentences were changed to probation following exposure to the operational costs and the prompt to justify incarceration in light of these costs (see Table 1 and Figure 1). This test was repeated after excluding two potential outlier cases and after log-transforming our dependent measures to approximate a normal distribution. However, the overall pattern remained unchanged (all *p*'s < 0.01). Self-reported political ideology was not associated with any of these effects (see Appendix B for details).

**Table 1:** Percentage of offenders sentenced to prison.

**Justification prompt**	**Prison capacity information**			
	**Absent**		**Present**	
	***M*** **[*****CI*****]**	***SE***	***M*** **[*****CI*****]**	***SE***
Before	75.89 [71.82, 79.97]	2.07	66.24 [62.10, 70.34]	2.10
After	69.71 [65.14, 74.28]	2.32	51.13 [46.50, 55.77]	2.35

Mean percentage (*M*) of offenders sentenced to prison with 95% confidence interval (*CI*) and standard error (*SE*). All comparisons are statistically significant at *p* < 0.001.

As a test of the robustness of these effects, we repeated our hypothesis tests in two subsamples (*N*_1_ = 134; *N*_2_ = 179), distinguished by the two semesters in which the data were collected. For each comparison, the pattern of significance persisted in both subsamples, indicating that the observed effects are robust to variation in sample size and composition (see Appendix B for subsample results).

### Exploratory analysis

Next, we assessed whether participants explicitly endorsed the belief that judges should consider capacity limits in their sentencing decisions. If they agreed with this belief, it could potentially explain why their sentences were so receptive to the capacity information. On average, they did express agreement with the belief, one-sample *t*_(213)_ = 1.81, *p* = 0.04, *M* = 0.23, *SE* = 0.13. However, the effect was small (Cohen's *d* = 1.89), due in part to wide variability. Interestingly, when we repeated our original hypothesis test, only including the data from participants who did *not* agree that judges should consider capacity limits, our original hypotheses remained fully supported. For those individuals, exposure to the justification prompt reduced incarceration rates [*M* = 62.30%, *SE* = 2.37, 95% *CI* (57.60, 67.00)] relative to that obtained before the prompt [*M* = 70.98%, *SE* = 2.07, 95% *CI* (66.87, 75.09)], *F*_(1, 108)_ = 32.20, *p* < 0.001, $\eta_{\text{p}}^{2}$ = 0.230. A main effect of capacity information was also obtained, *F*_(1, 108)_ = 9.70, *p* = 0.002, $\eta_{\text{p}}^{2}$ = 0.082. Incarceration rates were lower among participants who were exposed to the capacity information [*M* = 60.13%, *SE* = 2.76, 95% *CI* (54.65, 65.60)] than those who were not [*M* = 73.15%, *SE* = 3.14, 95% *CI* (66.93, 79.37)]. These main effects were qualified by an interaction, *F*_(1, 108)_ = 7.01, *p* = 0.000, $\eta_{\text{p}}^{2}$ = 0.061. Incarceration rates were lowest when participants were exposed both to the capacity information and the justification prompt [*M* = 53.76%, *SE* = 3.13, 95% *CI* (47.56, 59.97), *p* < 0.001] compared to when they were exposed to the capacity information [*M* = 66.49%, *SE* = 2.74, 95% *CI* (61.06, 71.92), *p* < 0.001] or the justification prompt alone [*M* = 70.83%, *SE* = 3.56, 95% *CI* (63.78, 77.89), *p* = 0.046]. These incarceration rates were lower than observed when participants were not exposed to either manipulation [*M* = 75.46%, *SE* = 3.11, 95% *CI* (69.29, 81.63)]. This replication of our effects suggests that the reductive effect of the capacity information on incarceration rates is not limited to those who expressly agree with its intent. Rather, it has a mitigating effect even among those who do not claim to agree that judges should consider capacity constraints.

At the individual crime level, we expected that people would be more likely to change their initial prison sentences to probation for the crimes that were less serious and that such changes would be greater following exposure to the prison capacity constraint. Consistent with expectation, participants were much more likely to change their initial prison sentence to probation when the crime was less serious (see Table 2, column B), as judged by their initial sentencing recommendations (column A), *r* = −0.81, *p* = 0.008. Critically, most of those decision changes were precipitated by exposure to the prison capacity constraint (column C). The only clear exception was for murder, which validates our prediction that changes from prison to probation would prioritize the less serious crimes.

**Table 2:** Incarceration rates as a function of crime type and prison capacity information.

**Crime**	**(A) Percentage who recommended prison initially**	**(B) Percentage of “A” who changed from prison to probation**	**(C) Ratio of “B” exposed to prison cap vs. not exposed**
Burglary	41.6	31.5	23.6 : 7.9^***^
Tax fraud	44.9	24.0	15.6 : 8.3^*^
Simple battery	64.5	10.9	8.7 : 2.2^***^
Drug trafficking	68.2	13.0	8.9 : 4.1^(0.055)^
Insurance fraud	68.7	26.5	19.0 : 7.5^***^
Assault and battery	81.3	14.9	10.3 : 4.6^**^
Armed robbery	82.2	12.5	9.1 : 3.4^**^
Agg. robbery	86.4	7.6	5.4 : 2.2^(0.057)^
Murder	87.4	7.0	3.7 : 3.2

*p*-value,

* *p* < 0.05,

** *p* < 0.01,

*** *p* < 0.001. Marginally significant values noted in parentheses.

An analysis of philosophical punishment justifications (retributive vs. utilitarian) indicated that self-identified retributivists chose incarceration at significantly higher rates [*M* = 72.86, *SE* = 2.89, 95% *CI* (67.17, 78.55)] than self-identified utilitarians [*M* = 63.80, *SE* = 1.83, 95% *CI* (60.20, 67.41)]; *F*_(1, 172)_ = 7.04, *p* = 0.009, $\eta_{\text{p}}^{2}$ = 0.039. However, there were no interactions between punishment justification and capacity information (*p* = 0.85) or justification prompt (*p* = 0.76).

## Discussion

This study demonstrates that mere exposure to a brief informational message about prison capacity (without any fixed capacity limitation) may be sufficient to reduce non-experts' decisions to incarcerate, and does so even more strongly when respondents are prompted to justify their decisions. Participants exposed to our manipulations willingly changed their prison sentence recommendations to probation for 1 in 4 defendants, primarily for the less serious crimes (e.g., burglary and insurance fraud). This fact is impressive considering that all nine crimes would typically qualify for incarceration in most jurisdictions, and in our study, even the least serious crime (burglary) still garnered a large percentage (~ 42%) of participants recommending prison initially.

Our results are consistent with our hypotheses and support policy arguments made by Jonson et al. (2014), Bierschbach and Bibas (2017), and others that simply asking judges to justify overages in prison capacity could reduce their reliance on incarceration by prompting judges to internalize the costs. Such a policy would seem to require minimal, if any, statutory revision since trial judges are already expected to provide justifications for many of their decisions. Even the Model Penal Code now states that the purposes of punishment include “to ensure that adequate resources are available for carrying out sentences imposed” and “to increase the transparency of the sentencing and corrections system, its accountability to the public, and the legitimacy of its operations as perceived by all affected communities” (§1.02, American Law Institute, 2017).

Our findings also comport with previous research on sentence length determinations that, by default, people tend to neglect to consider the costs of punishment, assuming a *de facto* policy to punish at any cost. But this cost neglect can be minimized by making these costs explicit (Rachlinski et al., 2013; Gottlieb, 2017; Aharoni et al., 2019, 2020, 2021, 2022). One explanation for this reversal is that making the costs salient leads decision makers to switch from a strategy of maximizing the benefits of their punishments to a strategy of balancing those benefits against the costs, as has been argued elsewhere (Aharoni et al., 2021). The present findings extend this scholarship by showing, for the first time, that (1) justification prompts alone are sufficient to reduce incarceration rates, (2) increasing the salience of operational prison costs (i.e., capacity considerations) is also sufficient to reduce incarceration rates, and (3) incarceration rates are most strongly reduced when decision makers are asked to justify their sentences *with respect to* these expected costs. This cost disclosure also reduced the expression of retributive sentencing justifications for punishment by a wide margin (~40%), as shown in our Supplementary material (see Appendix B for qualitative results).

The observed effect on incarceration decisions could not be fully explained by participants' expressed attitudes about whether, as a matter of policy, judges should be allowed to consider prison capacity in their sentencing judgments. Even those who did not expressly support such a policy reduced incarceration following exposure to our manipulations. This pattern suggests that such policies, much like a nudge, could exert their intended effects regardless of the decision maker's explicit views on the matter.

The fact that a justification prompt alone (without capacity information) was sufficient to reduce participants' incarceration rates is remarkable in its own right. Two plausible explanations come to mind. First, the justificatory act may prompt decision makers to consider their reasons more deeply, and this greater depth of processing might result in consideration of new reasons and different conclusions. Evidence on decision making and persuasion aligns well with this interpretation (see literature on Decision Justification Theory, Connolly and Zeelenberg, 2002; and justification pressure, Huber and Seiser, 2001). Second, the act of justification is effortful, and decision makers, consciously or unconsciously, may change their choice in order to economize on effort. This possibility could be addressed by a design that requires participants to justify all of their choice options including prison and probation.

Most retributive theorists and legal professionals recognize that the moral and societal benefits of punishing convicted criminals in proportion to the harms they caused should not be the only consideration in sentencing, and should be balanced with other considerations, such as deterrence, rehabilitation, and even financial costs. Hence, if juries and judges who sentence criminals are psychologically predisposed to neglect these consequentialist factors, nudging them in various ways may help them to consider factors they accept as relevant to such decisions. Our results suggest that such nudges can be effective. It would also be important to test the degree to which decision-makers believe their judgments in light of such information better accord with their considered views of how to balance various factors when punishing offenders.

This study represents an early proof of concept to motivate follow-on research on this topic. The undergraduates in our study, of course, are not professional judges, the vignettes they read included much less information than judges consider in actual criminal cases, and their decisions were purely hypothetical. Judges' extensive training in the law, their individual sentencing philosophies, and their professional motivation to take sentencing seriously, among other things, might make them less susceptible to the effects observed in this study. However, judges are not immune to the influence of contextual factors, and studies have demonstrated many parallels between judicial and non-expert punishment judgments (see Guthrie et al., 2000; Robbennolt, 2005; Teichman and Zamir, 2014; Miller, 2019). So replicating the current study with judges would represent an important further contribution to the field. If judges' punishment decisions are affected in ways that are similar to the participants in the present study, the impact could be substantial. Given the scale of mass incarceration in the U.S. today, diverting one in four of the least serious offenders to probation, or other alternatives to incarceration such as halfway houses, mediation or restorative justice programs, would represent a major savings in incarceration costs, not to mention the many intangible benefits such as helping defendants to maintain employment and keeping their families together. Understanding the punishment attitudes of non-experts is also important in its own right because these attitudes influence civic engagement, including the people and policies for which citizens vote.

One implication of our study is that there would be no need to mandate that judges reduce their incarceration rates–so-called “hard caps;” they would do so using their own discretion, by virtue of a simple change to their choice architecture: a brief instruction to justify any overages in capacity. The nature of that justification may be less relevant, since the request alone may be sufficient to motivate change. And since judges are already accustomed to requirements to justify their legal decisions in other contexts (such as in bench trials and rulings on motions), the transition cost of such a policy is likely to be minimal. Similar arguments could be made for prosecutors, who are tasked to make sentencing recommendations to judges, and for defense lawyers, who are tasked to negotiate for mitigation via plea agreements and other mechanisms. The degree to which these groups are responsive to punishment cost salience could be decisive for their case strategies and the subsequent incarceration rates. Before such a policy would be adopted, much work would be needed, not only to validate our behavioral predictions, but to assess legal and procedural feasibility of implementing the policy and tracking its impacts across branches of government. Therefore, testing the generalizability of these findings—both experimentally and naturalistically—to professional judges, prosecutors, and defense lawyers represents a promising area of future research.

## Data availability statement

The datasets presented in this study can be found in online repositories. The names of the repository/repositories and accession number(s) can be found below: https://osf.io/sp983/.

## Ethics statement

The studies involving human participants were reviewed and approved by University Research Services and Administration, Georgia State University. The patients/participants provided their written informed consent to participate in this study.

## Author contributions

EA, EN, and MH contributed to conceptualization and methodology. EA conducted the analyses with assistance from SF and prepared the original draft of the manuscript. SF assisted with management of research assistants. All authors contributed to manuscript revisions. All authors contributed to the article and approved the submitted version.

## Footnotes

^1^Three other measures were also administered as part of an unrelated study: the Leary et al. (2017) Intellectual Humility scale, internally developed items for measuring implicit motives for punishment, and a question on the extent to which participants believed their sentence recommendations differed from those of their peers.
