# Supplementary material for: Punishment as a scarce resource: a potential policy intervention for managing incarceration rates
Source: Front Psychol. 2023 May 5;14:1157460. doi: 10.3389/fpsyg.2023.1157460 (PMC10196492; doi:10.3389/fpsyg.2023.1157460)
Supplement: Supplementary file 1 [file Data_Sheet_1.DOCX]

**Appendix A**

**Survey Instructions**

The initial instruction was presented as follows, with the manipulated text (present vs. absent) in brackets.

Imagine you are a judge. You will read case summaries about 9 people who committed different crimes. Then you will make sentencing decisions. Last, you will be asked questions about yourself and about the cases. You may not go back to previous pages. You may be quizzed about the details of some of the crimes, so please read carefully.

[Today, there are 9 convicted felons on your schedule to be sentenced. But the prisons in your jurisdiction have recently reached full capacity. You may exceed the capacity by sentencing any of these felons to prison, but doing so is very costly. Therefore, the state legislature requires that for every offender that you sentence to prison, you must provide a written justification for why the recommended term of incarceration is worth the additional costs. Offenders who are not sentenced to prison will be placed on probation.]

Please read all 9 cases before answering any questions. Then for each crime, decide whether the offender should be incarcerated in prison for his offense, keeping in mind that you must provide a justification for each offender that you sentence to prison. Offenders who are not incarcerated will be placed on probation.

**Survey Stimuli**

Andrew forced his way into a suburban home at gunpoint. Threatening the family, he made them hand over cash, jewelry, and electronic devices worth approximately $12,000. He left without harming the family. Andrew was convicted for the crime of *Armed Robbery*.

Chris recently finished distributing a shipment of 5 kilograms of crack-cocaine to drug dealers in his neighborhood. Chris was convicted for the crime of *Drug Trafficking*.

While a family was on vacation, Seth jimmied the back door to their house and stepped into their kitchen. On the counter, he saw a new laptop computer, which he carried away. Seth was convicted for the crime of *Burglary*.

Jake traveled around residential communities selling a fraudulent health insurance supplement that never paid out to poor elderly residents. He defrauded an estimated 90 people in this fashion for anywhere from $350 - $900 each. Jake was convicted for the crime of *Insurance Fraud*.

Angry after overhearing another parent’s remarks during a soccer match in which Doug's son was playing, Doug approached the man after the game, grabbed his coffee mug, knocked him down, then kicked him several times while he was on the ground, knocking him out for several minutes and causing cuts that required five stitches. Doug was convicted for the crime of Battery.

As a woman searched her purse for car keys in a mall parking lot, Mike ran up and grabbed her gold necklace but it did not break. He yanked the woman to the ground by her necklace, where she gashed her head, requiring stitches. He ran off without the necklace. Mike was convicted for the crime of Assault and Battery.

To force a man to give up his wallet during a robbery attempt, Joe beat the man until he relinquished his wallet, which contained $350. The man was hospitalized for two days. Joe was convicted for the crime of *Robbery*.

Frank is a self-employed businessman. He deliberately underreported his earnings and overreported his expenses to pay less in taxes. He has avoided paying $19,000 in taxes by doing this. Frank was convicted for the crime of *Tax Fraud*.

Bill was running errands at a shopping mall but had to baby-sit his friend’s toddler. It occurred to him that it was too hot to safely leave the toddler in the car but he decided to leave him anyway and to return soon. He got talking with the seller, however, and forgot about the toddler, who passed out and died. Bill was convicted for the crime of *Manslaughter*.

**Punishment Justification survey question**

The primary purpose of punishing criminals should be to:

- give them what they deserve, we condemn them for what they've done, and we restore moral order in the community.
- discourage them from committing more crimes, we discourage others from committing crimes, and we rehabilitate them.

**Appendix B**

**Supplemental Analysis**

**SA 1.** Collection of participant’s textual justifications for their prison sentences afforded an opportunity to qualitatively examine the relative prevalence of each justification type. Textual justifications were coded by a single trained rater, blind to the study hypotheses. Justifications were aggregated at the single participant level and classified as “retributive,” “utilitarian”, both of these, or none of these. Criteria for the retributive classification include references to any of the following: the offender deserved it; the punishment restores moral balance; the punishment is self-justified; “do the crime, do the time” sentiments; other retrospective aims. Criteria for the utilitarian classification included reference to: incapacitation; specific deterrence; general deterrence; rehabilitation; other prospective aims.

Only those who initially recommended incarceration were asked to explain their decision. About 10% of those (9.9%) were uninterpretable. Among those who provided an interpretable explanation, 72.0% cited at least one retributive justification, whereas only 22.9% cited at least one utilitarian justification, 14.0% cited both of them, and 19.2% cited neither. Incarceration rates, both before and after the justification prompt, were positively associated with the presence of retributive justifications, Pearson *r* (before) = 0.20, *p* = .004, *r* (after) = 0.30, *p* < .001, but not utilitarian ones, *r* (before) = -0.06, *p* = .39, *r* (after) = -0.03, *p* = .67. That is, people who more often favored incarceration were more likely to justify their decision on retributive grounds.

Retributive justifications varied widely in response to exposure to the capacity message, χ2 (1) = 41.96, *p* < .001. Over 90% (91.7%) of participants who were not exposed to the message cited at least one retributive justification (e.g., “This offender committed a violent crime and needs to be punished accordingly”), whereas only about half (51.9%) of the exposed group cited a retributive justification. By contrast, the prevalence of utilitarian justifications did not differ between the exposed (19.8%) and unexposed (25.9%) groups, χ2 (1) = 1.13, *p* = .29 (e.g., “he would possibly do it again”). This stark contrast suggests that increasing the salience of prison capacity costs dramatically shifted participants’ justifications for punishment away from retribution toward other justification types.

**SA 2.** We searched for possible effects of our prison capacity manipulation on sentence length recommendations. For each offender, participants were asked to indicate how long in prison he should be incarcerated for his offense on a ratio scale from 0 - 12 years in prison. We chose this single sentencing range to maximize experimental control, acknowledging that real-world sentencing ranges are much more varied across crimes and jurisdictions, but also more cumbersome with respect to drawing unbiased causal inferences. At first blush, one might expect that if a capacity message reduces the probability of incarceration, as we found in our main analysis, that message should also reduce the recommended length of incarceration. But when averaging the sentences across crimes, we found no evidence to support this prediction, *F*(1, 201) = 2.89, *p* = .09, *M(treatment)* = 5.36, *SE* = .24, 95% CI[4.89, 5.83], *M(control)* = 4.80, *SE* = .23, 95% CI[4.35, 5.26].^^[[1]](#footnote-1)^^ See Table 2 for descriptive statistics.

**Table 2.**

| **Crime** | ***M*** | ***SE*** | **95% CI Lower** | **95% CI Upper** |
| --- | --- | --- | --- | --- |
| Burglary | 3.60 | 0.43 | 2.71 | 4.49 |
| Simple battery | 3.98 | 0.58 | 2.80 | 5.17 |
| Tax fraud | 4.31 | 0.61 | 3.06 | 5.57 |
| Insurance fraud | 5.39 | 0.46 | 4.44 | 6.33 |
| Aggravated robbery | 5.70 | 0.56 | 4.56 | 6.85 |
| Assault & battery | 6.20 | 0.58 | 5.00 | 7.40 |
| Drug trafficking | 6.40 | 0.69 | 4.98 | 7.82 |
| Murder | 10.42 | 0.53 | 9.33 | 11.51 |

*Note:* Mean (*M*) sentence length recommendations including standard error (*SE*) and confidence interval (CI) by crime type.

There are several possible reasons for this null effect on sentence length. One reason could be self-selection. That is, when participants resolved to incarcerate even after they were given an opportunity to reconsider, they might have been expressing a distinct motivation to punish that is impervious to compromise. Another possible reason is that the survey instructions did not explain how sentence length decisions would impact corrections budgets, only how placement decisions, namely prison vs. probation, would impact these budgets (i.e., number of people incarcerated at a given time). It might require an extra inferential leap to recognize how one offender’s prison term length could affect overall prison capacity, and participants might not have made that leap. A third reason could be that the sentence length decisions only appeared after all of the decisions to incarcerate were made, so in that time, participants could simply have forgotten the message about prison capacity. A fourth reason could be that our use of a standardized scale for all crimes could have reduced the probability of detecting a true effect because of range restriction. For all of these reasons, caution is warranted against over-interpretation of the sentence length result.

**SA 3.** We tested whether the effects of our hypothesis tests were associated with political ideology. They were not. In a repeated-measures ANOVA with political ideology entered as a covariate, there was no main effect of political ideology (*p* = .29), and political ideology was not associated with incarceration rates before vs. after the justification prompt (*p* = .99). However, the original interactive effect of cost information and justification prompt was preserved, controlling for political ideology, *F*(1, 195) = 12.81, *p* < .001.

**SA 4.**

As a test of the robustness of these effects, we repeated our hypothesis tests in two subsamples (N_1_ = 134; N_2_ = 179), distinguished by the two semesters in which the data were collected. For each comparison, the pattern of significance upheld among both subsamples, indicating that the observed effects are robust to variation in sample size and composition.

For N_1_ = 134, justification prompt exerted a main effect on incarceration rates, *F*(1, 82) = 37.31, *p* < .001, η_p_^2^ = 0.313. Specifically, for those who initially recommended incarceration (*M* = 74.00%, *SE* = 2.25, 95% CI[69.51, 78.47]), exposure to the justification prompt evoked a 8.70% reduction in their incarceration rate (*M* = 65.30%, *SE* = 2.36, 95% CI[60.60, 70.01]). This suggests that a justification prompt alone is sufficient to reduce the incarceration rate, consistent with predictions. A main effect of capacity instruction was also obtained, *F*(1, 82) = 9.78, *p* = .002, η_p_^2^ = 0.107. Incarceration rates were (13.74%) lower among participants who were exposed to the capacity instruction (*M* = 62.78%, *SE* = 3.18, 95% CI[56.45, 69.10]) than those who were not (*M* = 76.52%, *SE* = 3.03, 95% CI[70.49, 82.55]). These main effects were qualified by an interaction, *F*(1, 82) = 10.68, *p* = .002, η_p_^2^ = 0.115. Incarceration rates were lowest when participants were exposed both to the capacity instruction and the justification prompt (*M* = 56.11%, *SE* = 3.42, 95% CI[49.30, 62.92], *p* < .001) compared to when they were exposed to the capacity instruction alone (*M* = 69.44%, *SE* = 3.26, 95% CI[62.96, 75.93], *p* = .047) or the justification prompt alone (*M* = 74.50%, *SE* = 3.26, 95% CI[68.01, 80.99], *p* = .043). These incarceration rates were 22.43% lower than obtained when participants were not exposed to either manipulation (*M* = 78.54%, *SE* = 3.12, 95% CI[72.35, 84.72]).

For N_2_ = 179, we successfully replicated the main effect of justification prompt, *F*(1, 123) = 50.82, *p* < .001, η_p_^2^ = 0.292. For those who initially recommended incarceration, exposure to the justification prompt reduced incarceration rates (*M* = 69.11%, *SE* = 1.94, 95% CI[65.27, 72.94]) by 11.97% (*M* = 57.14%, *SE* = 2.23, 95% CI[52.72, 61.55]). We also replicated the main effect of capacity instruction, *F*(1, 123) = 13.57, *p* < .001, η_p_^2^ = 0.099. Incarceration rates were 14.08% lower among participants who were exposed to the capacity instruction (*M* = 56.09%, *SE* = 2.69, 95% CI[50.76, 61.44]) than those who were not (*M* = 70.16%, *SE* = 2.71, 95% CI[64.79, 75.53]). Again, these two variables interacted, *F*(1, 123) = 6.44, *p* = .012, η_p_^2^ = 0.050, such that incarceration rates were lowest when participants were exposed both to the capacity instruction and the justification prompt (*M* = 47.97%, *SE* = 3.14, 95% CI[41.76, 54.18], *p* < .001) compared to when they were exposed to the capacity instruction alone (*M* = 64.20%, *SE* = 2.73, 95% CI[58.80, 69.60], *p* = .012) or the justification prompt alone (*M* = 66.31%, *SE* = 3.16, 95% CI[60.05, 72.57], *p* = .002). These incarceration rates were 26.04% lower than observed when participants were not exposed to either manipulation (*M* = 74.01%, *SE* = 2.75, 95% CI[68.57, 79.46]), slightly exceeding the reduction obtained in study 1.

1. Scores for armed robbery were not included in the sentencing length analysis as a result of missing data from a programming error. [↑](#footnote-ref-1)
